# The influence of perceived threat on the motive attribution asymmetry bias for groups in conflict

**DOI:** 10.1371/journal.pone.0330927

**Published:** 2025-09-04

**Authors:** Rebecca E. Hughes, Brandon D. Stewart

**Affiliations:** 1 University of Birmingham, United Kingdom; 2 University of Southern Maine, United States of America; Goethe University Frankfurt am Main, GERMANY

## Abstract

Previous research shows higher perceived threat is related to more intergroup bias, usually via greater ingroup positivity. Newer research has identified the Motive Asymmetry Attribution Bias in which ingroup and outgroup members make very different explanations for the motives about why their groups are in conflict. We were interested in this Motive Asymmetry Bias and its relationship to perceived threat with groups in conflict, so we designed two studies to investigate it cross-sectionally (Study 1) and longitudinally (Study 2). We recruited samples of American Republicans and Democrats to complete an online survey measuring perceived threat and Motive Asymmetry Bias. Regression analyses indicated that perceived threat was not related to ratings of one’s own party; however, higher perceived threat was related to more negative ratings of the other party. This discovery is important to help inform different ways to intervene to improve intergroup relations, especially for groups in conflict.

## Introduction

When considering interactions with members of our own group and those of other groups, how do we define and view our motives and behaviors? Do we view others’ motives and behaviors positively, negatively, or a mixture of the two? Overall, research within intergroup relations has established a general intergroup bias, where the ingroup and outgroup are viewed differently [1,2]. Regardless of the group(s) to which individuals identify or belong, the ingroup is typically favored over the outgroup, which has been identified as Intergroup Bias or Ingroup Bias within the literature. It is, however, also essential to consider factors that may influence this intergroup bias, such as (a) whether ingroup favoritism, outgroup derogation, or both function as the driving force of the bias and (b) whether perceived threat influences people’s explanations for bias, especially when groups are in conflict.

Research has demonstrated that intergroup bias tends toward a preference for the ingroup without perceived negativity toward the outgroup [3,4], yet whether group members recognize this bias depends on whose point-of-view is being examined. The perception of intergroup bias may differ between the people observing it, either an ingroup or outgroup member, and from where the bias seems to be emanating. An individual may show intergroup bias where they perceive biased perceptions in the outgroup, but do not perceive themselves to be biased [5,6]. These effects demonstrate an asymmetry of perception of the ingroup and outgroup for intergroup bias – from the ingroup’s perspective, the outgroup is capable of biased judgments, yet the ingroup is not perceived to show this biased judgment. Thus, it may be that this asymmetry extends to perceptions of the outgroup as being biased against the ingroup without the ingroup recognizing its own self-preference and potential bias against the outgroup. We are interested in exploring how threat may relate to asymmetry in views because threat has been shown to be one of the major factors in altering intergroup views and may play a role in perceptions of motives for groups in conflict [7–10].

Waytz et al. [11] identified an effect on motives similar to the effects described above for intergroup bias, but they observed it specifically for groups who have experienced conflict; they named this phenomenon the Motive Attribution Asymmetry Bias (referred to hereafter as the “Motive Asymmetry Bias”), which is a difference in the attribution of motives for conflict made by one’s own group (ingroup) and the other group (outgroup). The ingroup tends to attribute their own reasons for conflict to ingroup love (i.e., showing ingroup favoritism), and the outgroup’s reasons to outgroup hate (i.e., outgroup derogation of the ingroup). However, when these motives are examined from the outgroup’s point of view, the outgroup is also saying that conflict is occurring between groups because their group is showing positivity toward their group, but that they are not showing negativity toward our group (i.e., one’s ingroup). Neither group sees the similarity of their perceptions, and this can create problems with constructive dialog and interactions. We are interested in this phenomenon because, to date, the Motive Asymmetry Bias has not been replicated and has not been investigated in relation to perceived threat. Because of perceived threat’s influential role in altering intergroup bias between groups, understanding this relationship could be important for reducing the Motive Asymmetry Bias, reducing conflict, and improving dialogs between groups.

While there are a few frameworks to choose from, we focus on perceived realistic and symbolic threat and do so within Intergroup Threat Theory framework because intergroup threat is central to groups in conflict. Stephan and Stephan [12] developed the Intergroup Threat Theory (ITT), which explores and defines the relationships between intergroup threat and intergroup bias. This theory highlights realistic threats (i.e., threats to a group’s resources) and symbolic threats (i.e., threats to a group’s values), and notes that negative stereotypes are antecedents to threat while intergroup anxiety is a subtype of realistic and symbolic threat [13–17]. Situations and expectations can increase these threat perceptions directly or they could increase negative stereotypes or intergroup anxiety, which can feed into and exacerbate threat perceptions. Intergroup Threat Theory hypothesizes that increased perceptions of symbolic and/or realistic threats can increase negative attitudes toward the outgroup viewed as the source of the threat, while less is known about how it alters perceptions of the ingroup [18].

We were interested in whether or in what way perceived threat may relate to the Motive Asymmetry Bias for groups in conflict. By examining the association with threat, we will have a better understanding of this bias and ways to potentially reduce it. Moreover, because this bias includes the ratings of both ingroups and outgroups, investigating it has the potential to significantly extend research on ingroup bias and outgroup derogation as mechanisms of the Motive Asymmetry Bias for groups in conflict and whether both forms of bias drive this effect. Yet, much of this research does not examine the separate influences of Ingroup Favoritism or Outgroup Derogation, nor does it focus on political groups in conflict or the Motive Asymmetry Bias [19,20,21,22]. We designed studies to examine the relationship of perceived threat with the Motive Asymmetry Bias between groups in conflict – specifically American Republicans and Democrats because they were one of the group used in the original studies by Waytz and colleagues [11].

Intergroup bias can occur in several different ways. It can be for the ingroup (ingroup favoritism), against the outgroup (outgroup derogation or hostility), or a mixture of the two [7,23]. We investigated these three hypotheses to explain how perceived threat may be associated with the Motive Asymmetry Bias: Ingroup Favoritism only, Outgroup Derogation only, and Combined Favoritism-Derogation effect (both favoritism and derogation), which we will review in turn.

### Threat related to only ingroup favoritism

Much of the research on intergroup relationships indicates that intergroup bias may be motived by ingroup favoritism rather than outgroup derogation [2]. Halevy, Weisel, and Bornstein [24] utilized the Intergroup Prisoner’s Dilemma-Maximizing Differences game, developed to distinguish between ingroup favoritism and outgroup derogation. They found that group members cooperated with their ingroup instead of competing with and derogating the outgroup for relative standing; this effect occurred even in the repeated games conditions that happened after conflict was repeatedly experienced between the groups. Other research has found that ingroup favoritism occurs more frequently in the minimal group paradigm, though there is an asymmetry in which favoritism is greater when people make positive evaluations or allocations of resources than for negative evaluations or allocations [25,26]. Several reviews of the literature also support the general finding of more ingroup favoritism. Greenwald and Pettigrew [3] reviewed different methodologies and research and found ingroup favoritism to be more prevalent than outgroup hostility in discrimination. Balliet et al. [1] conducted a meta-analysis and observed that intergroup discrimination in cooperative decision-making situations was due to ingroup favoritism instead of outgroup derogation. According to Social Identity Theory (SIT), ingroup members are motivated to perceive the ingroup positively, and strive not only for differentiation from the outgroup, but positive distinctiveness for their ingroup [7,27]. When one’s social group conflicts with another group or is experiencing social threat, a person may be more likely to associate with their social group if it is perceived as positive. One major strategy to achieve this can be to enhance their group through ingroup favoritism. Thus, those individuals who perceive high threats to their group may rate their ingroup’s motives for conflict as more positive while ignoring the outgroup’s motives (Ingroup Favoritism hypothesis).

### Threat related to only outgroup derogation

Other research indicates that threats may relate more to negative feelings against the outgroup [21,28]. However, when these ideas are extended to examining groups in conflict, the results change. Most of the research on groups in conflict shows that ingroup favoritism is still the main motivation for individuals instead of outgroup derogation [4,8,9]. There are, however, noted exceptions. Branscombe and Wann [29] observed that outgroup derogation occurred when the ingroup was threatened by a group with which they were in conflict, but derogation did not occur when it was not threatened, and that derogation was greatest for those with lower collective self-esteem. Greenwald and Pettigrew [3] hypothesized that negative outgroup ratings should be more likely in protracted conflicts with a long history of intergroup discrimination such as Northern Ireland and South Africa. These area and country differences highlight other possibilities for the role of conflict that may have been missed in previous research. Importantly, much of the research on conflict has been done with artificial groups, which may not possess the enmity that is often seen with actual groups in conflict, or the research has been done with blatant outgroup derogation instead of subtle outgroup derogation: Weisel and Böhm [30] examined natural groups who had high animosity (e.g., fans of sports teams and supporters of political parties) and they manipulated whether harm to the outgroup was blatant (actively harming) or was subtle (withholding money). When harming the outgroup was active or blatant, then ingroup favoritism was more likely, but when harming the outgroup was passive or subtle, then outgroup derogation was more likely. This effect, however, was more reliable and robust when groups had high animosity and moral differences (political groups); without the moral differences, but with high animosity (sports teams), ingroup favoritism occurred only half the time.

This research aligns with research within political psychology. Abramowitz and Webster [31] have noted a change in American politics in the last 40 years. They found that there has been an increase in negative partisanship, which is defined as “the phenomenon whereby Americans largely align against one party instead of affiliating with the other” (p. 119). In their analysis of the American National Election Study data, they found that the rating of one’s own party was stable on the 0 (very cold or unfavorable feeling) to 100 (very warm of unfavorable) feeling thermometer (e.g., mid-70s ratings in 1988 to mid-60s in 2016) while the rating of the other party has been low, but has decreased over the past three decades and remained low (e.g., mid-40s ratings in 1988 to low-20s in 2016). This research finding is supported by research on affective polarization, which is defined as partisans viewing each other as disliked outgroups, and that this affective polarization has been increasing over the last few decades [32]. There may be a greater amount of animosity for political parties that has remained relatively high for decades, which may change the nature of the response to those groups when in conflict. Taken together, these studies indicate that outgroup derogation may be more likely. Those individuals who perceive high threat may have associations of greater negativity and derogation of the outgroup without changing their view of the ingroup (Outgroup Derogation hypothesis).

### Threat related to ingroup favoritism and outgroup derogation

While perceived threat could be related to either ingroup favoritism or outgroup derogation separately, there is evidence that it could be related to both. Wlodarczyk et al. [33] noted that the best model of fit to explain prejudice in their study included both elements of ingroup favoritism and outgroup derogation – their model included prejudice mediating the relationship between threat and the response to the outgroup. This included showing that realistic threat was related to both more positive emotions toward the ingroup and more negative emotions in relation to the outgroup. Related more directly to our study design, Abbink and Harris [34] investigated in-group favoritism and out-group derogation in naturally occurring groups in a political conflict and in artificial groups. They observed ingroup favoritism in the artificial groups but observed both ingroup favoritism and outgroup derogation in the natural groups in conflict. Finally, the occurrence of both ingroup favoritism and outgroup hate may be more likely in situations where there is conflict or competition over resources or over political power [7,35]. Thus, there is enough evidence to also support a combination of ingroup favoritism and outgroup derogation [7,23]. Those individuals who perceive high threat may have stronger associations for both ingroup favoritism and outgroup derogation (Combined Favoritism-Derogation hypothesis).

### The current research

The previously reviewed research has noted some interesting effects in relation to animosity, morality, negative political partisanship, and affective polarization in which partisanship and polarization have been increasing over recent decades and these increases have occurred at the same time animosity between political groups has been high [31,32]. These relationships and the high perceived threat between the groups indicate that perceived threat would be maintained over time. However, little research has demonstrated a longitudinal link between perceived threat and intergroup bias over time [10,36–38]. The longitudinal research that does exist tends to connect perceived threat to various social ideologies or has examined unrelated types of threat such as stereotype threat [38,39]. To rectify this deficit, Study 1 sought to first examine the interaction between Perceived Threat and the Motive Asymmetry Bias at a single time-point while Study 2 sought to test it longitudinally. Within Study 2, we will use the same cross-sectional design used in Study 1, and then re-test participants three months later for the longitudinal component. We would predict that their level of perceived threat at time 1 should interact with the political party focus variable (own group versus other group) to predict motives at time 1 as well as at time 2.

Since this was a first investigation into the relationship between perceived threat and the Motive Attribution Asymmetry Bias, we used a well-established theory and measure of perceived threat. Specifically, we used perceived threats to the resources and symbolic values of one’s social group that Intergroup Threat Theory (ITT) argues are main sources of intergroup threat [16–18]. Given that we are using the Motive Asymmetry Bias paradigm, we were also able to investigate participants’ positive and negative views of their own group’s motives for conflict or participants’ positive and negative views of the other group’s motives. This measure therefore allowed us to estimate ingroup favoritism and outgroup derogation for motives in relation to perceived threat. Ethical approval was obtained for all studies through the University of Birmingham Ethical Review Committee. All data and research materials are available at https://osf.io/3SDYZ/.

#### Hypotheses.

***Hypothesis 1.*** *Ingroup Favoritism Effect*: We expect more positivity to be observed in ratings of one’s Own Political Party for those perceiving high threat compared to lower threat, but for there to be a non-significant difference of threat for the Other Party ratings.

***Hypothesis 2.*** *Outgroup Derogation Effect*: We expect more negativity to be observed in ratings of the Other Political Party for those perceiving high threat compared to low threat, but for there to be a non-significant difference of threat for Own Party ratings.

***Hypothesis 3.*** *Combined Favoritism-Derogation effect*: We would expect Own Political Party ratings to be more positive with higher perceived threat and Other Party ratings to be more negative with higher perceived threat compared to lower perceived threat.

## Study 1

### Methodology

#### Design.

We used a 2 (Party Focus: Own/Other Political Party) x 2 (Perceived Threat: High/Low) x 2 [Motive Rating: Love/Hate] mixed model design with manipulated Party Focus and measured Threat as between-participant factors and Motive as a within-participant factor. To control for any order effects, we counterbalanced the presentation of the Party Focus manipulation and the Perceived Threat measure so that each appeared first for fifty percent of the participants. Random assignment and counterbalancing produced four possible orders: (a) Threat first, then Own Party focus, (b) Threat first, then Other Party focus, (c) Own Party focus first, then Threat, or (d) Other Party focus first, then Threat. We followed previous online research and used the computer’s random number generator to randomly assign participants to conditions [40,41].

#### Participants.

We recruited six hundred sixty-six Democrats and Republicans via Prolific.com to participate in our study, recruitment and data collection were carried out in December 2018. Filtering procedures applied on the Prolific.com platform ensured that all participants were from the USA and over 18 years old. We first examined participants’ responses to two measures of political affiliation and removed any participants with responses that were misaligned with the stated party affiliation. Since the Republican party is associated with more conservative political views, and the Democratic party is associated with more liberal political views, participants’ data that showed a party misalignment (e.g., identified as Republican but extremely liberal on a Likert scale of (1) extremely liberal to (7) extremely conservative, or identified as Democrat but extremely conservative) were removed from the analyses. This procedure removed participants whose attitudes were ambivalent and who did not have a solid party identification. We had anticipated some loss of data due to misalignment, however, we were unsure of the expected effect sizes for this research because we were examining the relationship of threat to a novel effect. We conducted a priori power calculations using G*Power [42] that indicated a large sample of 650 participants to be able to observe very small effects (between *R*^2^ = .01 and.015) for the two-way interactions at 0.6 to 0.8 power. After removing these participants, data for 635 participants remained for analysis. Removal of these participants did not alter the results. Overall, participants were between 18 and 71 years old (*M *= 32.73, *SD* = 11.31) with 164 Republicans, 471 Democrats; 54.0% were female, and 75.6% were white. The current studies were not preregistered.

#### Materials.

**Perceived Threat from the Other Party Scale** Threat was measured using the Perceived Threat from the Other Party scale, which was adapted from Stephan, Boniecki, et al.’s [43] 24-item perceived Symbolic and Realistic Threat measure. This measure has been used in many research studies to measure perceived threat [44–49]. The scale consisted of twenty-four items that measured perceived threat based upon 12 symbolic threat items and 12 realistic threat items; all items were presented in a randomized order. The scale used a seven-point, vertical scale from (1) *Disagree Strongly* to (7) *Agree Strongly*. An example of the items included are “the other party holds too many positions of power and responsibility in this country,” and “my party has very different values than the other party” (S1 Appendix A). Because the perceived realistic and symbolic subscales share a common theme of threats to the ingroup [43,50] and because they were highly correlated (*r* = .68, *p* < .001), we used all twenty-four items in a single index of perceived threat. Single indexes of perceived threat have been used reliably in previous research [51–54]. After reverse-scoring items, scores were averaged and higher scored indicated more perceived threat from the other party (*M* = 5.04, *SD* = 0.81, *α* = .89).

**Love Composite Score (Ingroup Favoritism)** We used the same three items as Waytz et al. [11] for assessing positive motivations for being in conflict (i.e., empathy, compassion, kindness). For those in the own party condition, the questions followed a common format: “When your party engages in conflict, how much is your party motivated by empathy toward your political party?” For those in the other party condition, the questions were formatted in a similar manner: “When the other party engages in conflict, how much is their party motivated by empathy toward their political party?” Participants made their ratings on a 7-point Likert scale from (1) *Not at all* to (7) *Very Much* (S2 Appendix B). Ratings were averaged to create a positivity or Love composite score (*M* = 4.69, *SD* = 1.66, *α* = .95).

**Hate Composite Score (Outgroup Derogation)** We used the dislike and hatred items that Waytz et al. [11] had used, but we replaced their Indifference item with a Disdain item because they had found a low alpha reliability of only 0.59 with their composite of Dislike, Indifference, and Hatred items. They conducted their analyses with and without the Indifference item, which is more passive than dislike or hatred, and observed the same pattern and significance of results. Instead of using just a two-item measure, we added the Disdain item and kept the Dislike and Hatred items, which allowed us to retain the ability to do the analyses with just the two items if needed. For those in the own party condition, the questions were formatted in the following way “When your party engages in conflict, how much is your party motivated by hatred toward the other party?” For those in the other party condition, the questions were formatted such as “When the other party engages in conflict, how much is their party motivated by hatred toward your political party?” Participants made their ratings on a 7-point Likert scale from (1) *Not at all* to (7) *Very Much* (S2 Appendix B). Ratings were averaged to create a negativity or Hate composite score (*M* = 4.85, *SD* = 1.67, *α* = .92).

**Filler Task** Participants completed a filler task between the threat measure and intergroup bias measure to create a small separation between tasks. The four items (*M* = 3.58, *SD* = 0.88, *α* = .77) were from the Need for Cognition questionnaire [55] and were chosen because they have not correlated with the threat measure in past research [20] and did not correlate significantly in this study (*r *= .01, *p *= .762, S3 Appendix C).

**Political Affiliation** Participants completed two questions related to political affiliation that had been used by Waytz et al. [11]. One question asked them about the party to which they most closely identified with, Republican or Democrat (i.e., Political Alignment). All participants should have considered themselves a member of or aligned with one of these parties because we had selected only participants who had stated they aligned themselves with a party in their the Prolific.com recruitment survey. The second question asked the extent to which participants considered themselves as Liberal or Conservative (i.e., Political Ideology). They made their rating on a 7-point Likert scale ranging from (1) *Extremely Liberal* to (7) *Extremely Conservative* (*M *= 2.96, *SD *= 1.85; see S4 Appendix D).

#### Procedure.

First, participants provided written informed consent and then were randomly assigned to one of the four conditions. In the Own Party focus condition, participants answered questions about their party’s positive motives toward their own party and negative motives toward the other party. In the Other Party focus condition, participants answered questions about the other party’s positive motives toward the other party and negative motives toward the participant’s party. In between the Threat measure and Party Focus task, participants completed the four-item need for cognition filler task.

Participants then completed demographic questions, math filler questions, and additional filler questions of what they thought the purpose of the study was, their gender, ethnicity, age, and in what country they currently resided (S5 Appendix E). Next, we asked, “What political party do you feel more closely aligned with?” and then “Please rate your personal political orientation.” Finally, participants were debriefed.

## Results

### Motive Asymmetry Bias

**Fig 1:**
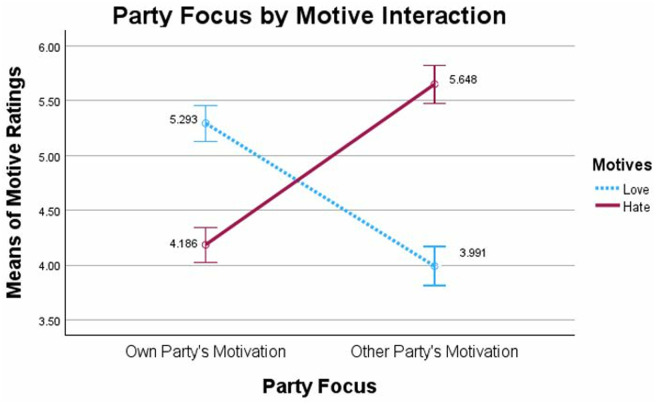
Motive Asymmetry Bias, Study 1: Ratings of motives for causing conflict as a function of rating their own political party’s motives or rating the other political party’s motives. Note: Significant Party Focus (Own, Other Party rated) by Motive (Love, Hate rated) interaction from mixed model ANOVA. Error bars show 95% confidence intervals.

Importantly, we replicated the Motive Asymmetry Bias findings from Waytz et al., 2014 [11]. The 2 (Party Focus: Own/Other Party) x 2 [Motive Ratings: Love/Hate] mixed model ANOVA with Motive as the within-participants factor revealed a significant and large interaction, *F*(1,633) = 219.997, *p *< .001, *η*²_p_ =.258 (see Fig 1). The data showed that when considering the ingroup’s motives for being in conflict, participants rated those motives more positively than the motives attributed to the outgroup.

### Party Focus x Perceived Threat on Relative Motive Attributions

**Relative Motive Attributions:** To conduct our analyses with a continuous variable (i.e., measured threat), we calculated an overall motive attributions score. *Relative Motive Attributions* were computed by taking the Love composite score and subtracting the mean Hate composite score. A higher and a numerically positive Motive Attribution Score would indicate more positive than negative motivations and a numerically negative score would indicate more negative than positive motivations.

**Fig 2:**
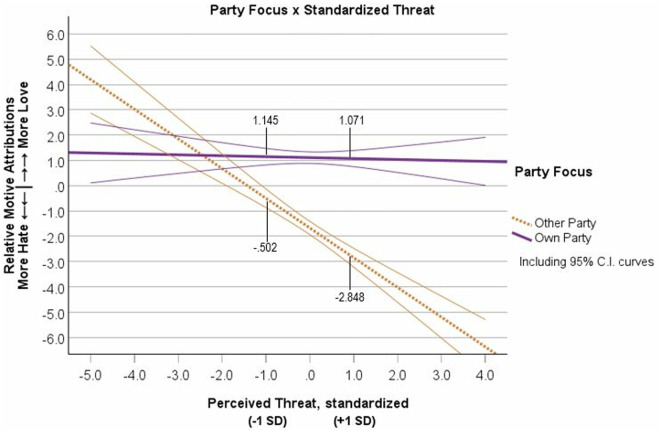
Study 1 ratings of relative motives (hate minus love) as a function of rating their own political party’s motives or rating the other political party’s motives and the level of perceived threat from the political outgroup. Note: Motive Attributions measured as Love minus Hate scores. Error curves show 95% confidence intervals.

To test whether Relative Motive Attributions were different in relation to Perceived Threat level or Party Focus, we effects-coded Party Focus (Own = 1, Other = −1) and standardized the continuous measure of Perceived Threat. We standardized the continuous predictor of Perceived Threat before creating the interaction term because failing to do so produces extreme multicollinearity between the continuous predictor and the interaction term, which interferes with the analysis and the interpretation of the interaction [56,57]. We entered effects-coded Party Focus, standardized Threat, and the interaction into a regression equation with Relative Motive Attributions (Motive Attribution Index = Love minus Hate scores) as the outcome (See table G1 in S7 Appendix G for correlations among all variables). The analysis revealed a significant main effect of standardized Threat, *semipartial R*^2^ = .069, *β* = −.222, *t* = −6.863, *p* < .001, *b* = −.605, 95% CI [−.778, −.432], and a main effect of Party Focus on Motive Attributions, *semipartial R*^2^ = .285, *β* = .511, *t* = 15.855, *p* < .001, *b* = 1.391, 95% CI [1.219, 1.564]. Notably, these main effects were qualified by a significant Party Focus x Threat interaction, *semipartial R*^2^ = .062, *β* = .208, *t* = 6.445, *p* < .001, *b* = .568, 95% CI [.395,.741], (see Fig 2). Results did not support an Ingroup Favoritism effect or the Combined Favoritism-Derogation effect. The Threat simple slope for the Own Party condition was not significant (*R*^2^ < .001, *β* = −.013, *p* = .760, *b* = −.037, 95% CI [−.273,.200]), but the Threat slope was significant for the Other Party condition (*R*^2^ = .116, *β* = −.430, *p* < .001, *b* = −1.173, 95% CI [−1.426, −.920]). This pattern of data supported only the Outgroup Derogation effect. Additionally, we found that separate analyses with symbolic and realistic threat did not alter the Party Focus x Threat results (see S10 Appendix J of the supplemental materials).

### Supplementary analyses

Like Waytz et al., 2014, Political Orientation also did not interact with our Party Focus x Threat interaction on Relative Motive Attributions [11]. We observed a non-significant Party Focus x Threat x Political Orientation interaction, *semipartial R*^2^ < .001, *β* = −.008, *t* = −.225, *p *= .822, *b* = −.019, 95% CI [−.184,.146]. The Party Focus x Threat interaction, however, remained significant, *semipartial R*^2^ = .054, *β* = .201, *t* = 5.971, *t* = 5.971, *p* < .001, *b* = .549, 95% CI [.369,.730], as did the main effect of Threat, *semipartial R*^2^ = .066, *β* = −.223, *t* = −6.628, *p* < .001, *b* = −.610, 95% CI [−.790, −.429], and main effect of Party Focus, *semipartial R*^2^ = .289, *β* = .509, *t* = 15.977, *p* < .001, *b* = 1.385, 95% CI [1.215, 1.555]. Thus, political ideology did not qualify the results.

We also tested for order effects by conducting a regression with effects-coded Party Focus, effects-coded Order (of Perceived Threat and Party Focus), standardized Perceived Threat, and all interaction terms in the model for Relative Motive Attribution. We observed a non-significant Party Focus x Threat x Order interaction, *semipartial R*^2^ = .004, *β* = .049, *t* = 1.504, *p* = .133, *b* = .133, 95% CI [−.041,.308], a non-significant Party Focus x Order, *semipartial R*^2^ = .001, *β* = .03, *t* = .941, *p* = .347, *b* = .083, 95% CI [−.090,.255], and non-significant Threat x Order interaction, *semipartial R*^2^ = .001, *β* = .023, *t* = .703, *p* = .482, *b* = .062, 95% CI [−.112,.237]; thus, order of presentation did not significantly change motive ratings.

## Discussion

Regression analyses did not support Hypothesis 1 (Ingroup Favoritism hypothesis) or Hypothesis 3 (Combined Favoritism-Derogation hypothesis) on Relative Motive Attributions. We, however, did observe support for Hypothesis 2 (Outgroup Derogation hypothesis). The Threat simple slope for Own Party Focus was non-significant, while the slope for Other Party Focus was significant, which shows more negativity in the Other Party condition for participants perceiving high threat compared to low threat. To support the Combined Favoritism-Derogation hypothesis, we would have expected that the Own Party focus slope would be significant and positive and that the Other Party focus slope would be significant and negative. To support the Ingroup Favoritism hypothesis, we would have expected only the Own Party focus slope to be significant. Neither of these hypotheses were supported. We conducted Study 2 to determine if we would replicate the observed Outgroup Derogation effect. In addition, we wanted to test if perceived threat were related to motive attributions over time, as some, but not much, previous research has suggested that threat may persist over time [31,32,58]. Thus, Study 2 also added a longitudinal aspect to investigate this idea.

Previous research on intergroup bias suggests the influence of social identity on intergroup bias is often explored within the Social Identity Theory framework [59,60]. Findings indicate that the amount one identifies with a group can influence intergroup bias [60,61], that social identity is related to perceived threats [18], and threat and identity could both influence intergroup bias [16]. With these considerations, we included a measure of social identification in Study 2 as an a priori variable to investigate if it related to threat.

## Study 2

### Hypotheses

We tested the same three hypotheses as in Study 1: The Ingroup Favoritism Effect (Hypothesis 1), the Outgroup Derogation Effect (Hypothesis 2), and the Combined Favoritism-Derogation effect (Hypothesis 3). We predicted that we would replicate the Outgroup Derogation hypothesis.

Based upon previous literature on social identification and perceived threat, we included a measure of social identification to test whether we would observe different relationships on the motive attributions and threat for those individuals who had high social identification with their party compared to low social identification with their party. We would expect there to be stronger relationships to threat for those with high identification (Hypothesis 5), but we were not confident that we would actually observe an interaction in this situation since Waytz and colleagues did not find that political/party orientation moderated their effects [11].

### Methodology

#### Design.

We used the same design as in Study, and random assignment and counterbalancing produced the same four orders for Time 1: (a) Threat first, then Own Party focus; (b) Threat first, then Other Party focus; (c) Own Party focus first, then Threat; (d) Other Party focus first, then Threat. There was a three-month gap between Time 1 and Time 2. We ensured that participants were assigned to the same Party Focus conditions as they completed in Time 1 in order to reduce suspicion. At Time 2, random assignment and counterbalancing again produced the same four orders.

Study 2 used the same measures of Perceived Threat, Motive Attributions, 4-item Need for Cognition, and Political affiliation as Study 1. The main difference was the inclusion of the longitudinal component and the inclusion of a Social Identification measure at the very end of the Time 1 and Time 2 parts of the Study.

#### Participants.

We recruited a total of 668 participants via Prolific.com, with recruitment and data collection in May 2019-August 2019. We once again removed participants that showed a party misalignment (e.g., identified as a Republican and extremely liberal or identified as a Democrat and extremely conservative). This left 641 participants’ data for analysis. Removal of these participants did not alter the results. Participants were between 18 and 74 years old (*M *= 34.79, *SD* = 18.45) with 277 Republicans and 364 Democrats; 51.0% were female and 85.2% were white. For the longitudinal analyses, we anticipated retaining approximately 60–70% of the sample at Time 2 (three months later) for a total of 390–455 participants. We used G*Power [42] to estimate observing a small effect size (*R*^2^ = .02) at 0.7 to 0.8 power for our two-way interaction analyses.

#### Materials.

**Social Identification** To measure social identification with the ingroup, we used four items that have been used extensively in previous studies to measure social identity [62–73]. The measure used a nine-point, vertical Likert scale from (0) *Not at all* to (8) *Very much* (S6 Appendix F). The four items were averaged, and higher scores indicated more social identification with one’s political group (*M* = 5.68, *SD* = 1.80, *α* = .91).

#### Procedure.

Participants provided written informed consent and were randomly assigned to one of the four, counterbalanced orders. After the main measures, participants completed the same filler questions from Study 1 that asked about mathematics problems, purpose of the study, gender, ethnicity, age, and in what country they reside. They next completed the same political affiliation questions used in Study 1, completed two additional need for cognition items, and then completed the Social Identification measure. Finally, they were debriefed.

Three months after completing Time 1 measures, participants completed the same measures at Time 2. To aid in the cover story and avoid suspicion, participants were ensured to be in the same Party Focus condition (either Own or Other) in Time 1 and 2.

## Results

### Motive Asymmetry Bias at Time 1

**Fig 3:**
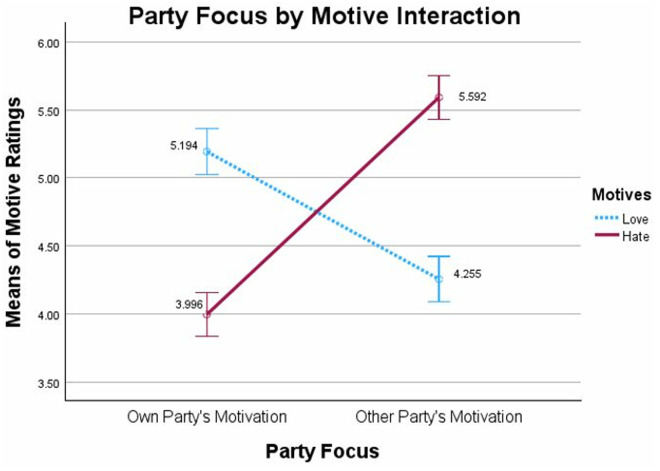
Motive Asymmetry Bias, Study 2 Time 1: Ratings of motives for causing conflict as a function of rating their own political party’s motives or rating the other political party’s motives. Note: Party Focus (Own, Other) by Motive (Love, Hate) mixed model ANOVA with motive rating as within-participants factor. Error bars show 95% confidence intervals.

We once again standardized the continuous predictor of Perceived Threat before creating the interaction term and analyzing the data from Time 1 to test whether we replicated the Motive Asymmetry Bias findings from Waytz et al. [11] and from Study 1. The 2 (Party Focus: Own/Other) x 2 [Motive Ratings: Love/Hate] mixed model ANOVA with Motive as the within-participants factor revealed a significant interaction, *F*(1,639) = 227.896, *p *< .001, *η*²_p_ =.263, which indicates a large Motive Asymmetry Bias (see Fig 3).

### Participants at Time 2 and Analysis of the Motive Asymmetry Bias at Time 2

Considering data from Time 2, we conducted the same analyses as those at Time 1. A total of 508 participants (79% of participants from Time 1) completed the study at Time 2 approximately three months after Time 1, with roughly the same number of participants in each condition. We again examined participants’ data for any discrepancy in responses to the two political affiliation measures. After removing participants with misaligned responses, 500 participants’ data (78%) remained for analysis at Time 2. Removal of these participants did not alter the results.

**Fig 4:**
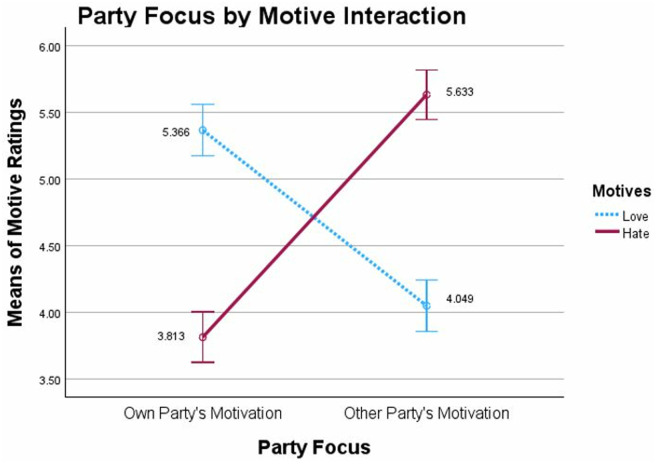
Motive Asymmetry Bias, Study 2, Time 2: Ratings of motives for causing conflict as a function of rating their own political party’s motives or rating the other political party’s motives. Note: Party Focus (Own, Other) by Motive rating (Love, Hate) mixed model ANOVA with Motive rating as within-subjects factor. Error bars show 95% confidence intervals.

We conducted a 2 (Party Focus: Own/Other) x 2 [Motive ratings: Love/Hate] mixed model ANOVA with Motive as the within-participants factor. This analysis produced a significant interaction, *F*(1,498) = 239.033, *p *< 0.001, *η*²_p_ = .324, which replicated both our previous findings and those of Waytz and colleagues (11) for a large Motive Asymmetry Bias (see Fig 4).

### Party Focus x Perceived Threat interaction on Relative Motive Attributions at Time 1

**Fig 5:**
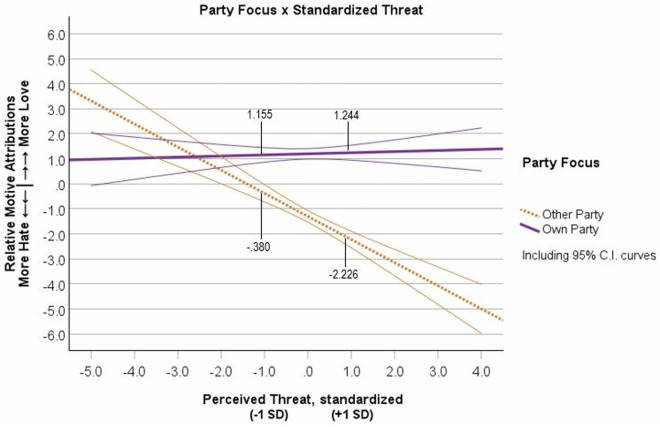
Study 2, Time 1 ratings of relative motives (hate minus love) as a function of rating their own political party’s motives or rating the other political party’s motives and the level of perceived threat from the political outgroup. Note: Motive Attributions measured as Love minus Hate scores. Error curves show 95% confidence intervals.

To test the interaction of Party Focus and level of Perceived Threat on Relative Motive Attributions, we entered effects-coded Party Focus (Own = 1, Other = −1), standardized Perceived Threat, and the interactions into a regression on Relative Motive Attributions at Time 1; see table G2 in S7 Appendix G for correlations among all variables of interest. We observed a significant main effect of standardized Threat, *semipartial R*^2^ = .045, *β* = −.178, *t* = −5.472, *p* < .001, *b* = −.439, 95% CI [−.597, −.282], and of Party Focus on Relative Motive Attributions, *semipartial R*^2^ = .277, *β* = .506, *t* = 15.603, *p* < .001, *b* = 1.251, 95% CI [1.094, 1.409]. Once again, we observed the important and significant Party Focus x Threat interaction that qualified these main effects, *semipartial R*^2^ = .054, *β* = .196, *t* = 6.025, *p* < .001, *b* = .484, 95% CI [.326,.642] (see Fig 5). The Threat simple slope for the Own Party condition was not significant (*R*^2^ < .001, *β* = .018, *p* = .691, *b* = .044, 95% CI [−.175,.264]), but the Threat slope for the Other Party condition was significant (*R*^2^ = .091, *β* = −.373, *p* < .001, *b* = −.923, 95% CI [−1.150, −.696]). These results supported the Outgroup Derogation effect that we had observed in Study 1 (Hypothesis 2). See S8 Appendix H for supplemental analyses showing non-significant effect of order of variables on the Party x Threat interaction, and non-significant interactions with political orientation.

### Party Focus x Perceived Threat interaction on Relative Motive Attributions at Time 2

**Fig 6:**
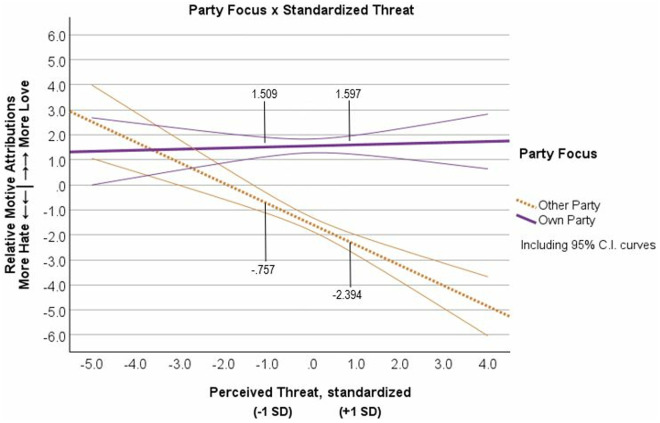
Study 2, Time 2 ratings of relative motives (hate minus love) as a function of rating their own political party’s motives or rating the other political party’s motives and the level of perceived threat from the political outgroup. Note: Motive Attributions measured as Love minus Hate scores. Error curves show 95% confidence intervals.

On the Time 2 data, we conducted the 2 (Party Focus: Own/Other) x 2 (Perceived Threat: High/Low) analysis on Relative Motive Attributions. We found a significant main effect of standardized Threat, *semipartial R*^2^ = .03, *β* = −.141, *t* = −3.917, *p* < .001, *b* = −.387, 95% CI [−.582, −.193] and of Party Focus, *semipartial R*^2^ = .337, *β* = .568, *t* = 15.878, *p* < .001, *b* = 1.564, 95% CI [1.371, 1.758], on Relative Motive Attributions. These effects were qualified by a significant Party Focus x Threat interaction, *semipartial R*^2^ = .037, *β* = .156, *t* = 4.359, *p* < .001, *b* = .431, 95% CI [.237,.626]. The Threat simple slope for the Own Party condition was not significant (*R*^2^ < .001, *β* = .016, *t *= .326, *p* = .745, *b* = .044, 95% CI [−.220,.308]), but the Threat slope was significant for the Other Party condition (*R*^2^ = .061, *β* = −.297, *t *= -.5.641 *p* < .001, *b* = −.819, 95% CI [−1.104, −.534]). These results supported the Outgroup Derogation effect (see Fig 6). See S8 Appendix H for supplemental analyses showing non-significant effects of order of variables and non-significant interactions with political orientation.

### Analyses with social identification at time 1 and at time 2

We had included social identification with one’s political party at the very end of the study to test whether there was a Party Focus x Perceived Threat x Social Identification interaction that would further clarify effects. We entered effects-coded Party Focus (Own = 1, Other = −1), standardized Perceived Threat, standardized Social Identification, and all interactions, including those with social identification, into the regression on Relative Motive Attributions at Time 1 (641 participants). We observed a significant Party Focus x Threat interaction, *semipartial R*^2^ = .028, *β* = .148, *t* = 4.286, *p* < .001, *b* = .366, 95% CI [.198,.534] that we have observed consistently. This interaction, however, was not qualified by a three-way Party Focus x Threat x Social Identification interaction on Time 1 data, *semipartial R*^2^ < .001, *β* = .006, *t* = .180, *p* = .857, *b* = .012, 95% CI [−.124,.149]. Additionally, for the Time 2 data, we also observed a non-significant Party Focus x Threat x Social Identification interaction, *semipartial R*^2^ = .005, *β* = −.079, *t* = −1.644, *p* = .101, *b* = −.109, 95% CI [−.174,.015]. Thus, Social Identification with political party did not explain or clarify our results in either Time 1 or Time 2 and we still observed a significant Party Focus x Threat interaction. This outcome was not entirely unexpected given that participants’ political orientation also did not moderate the Party Focus x Threat interaction in our Study 1 or in the Waytz et al. studies [11].

### Longitudinal analyses

There was a 78% completion rate for Time 2. This is a higher rate than is generally considered an acceptable retention rate for longitudinal studies, which is between 50 and 70% [74–76]. When designing the Time 2 portion of the study, we ensured that participants rated the same party (Own, Other) as in Time 1 and that the presentation of threat and motive measures were also counterbalanced at Time 2. We used this procedure to aid in the cover story and help make sure participants did not become suspicious of the true nature of the study.

The longitudinal design of this study serves to add an element of time to our examination of Perceived Threat and the Motive Asymmetry Bias. With the cross-sectional design of the study, we were able to gain information on the relationship between Perceived Threat and the Motive Asymmetry Bias in general. The longitudinal element added information on more chronic Perceived Threat and how it may relate to the Motive Attributions. This helps gain more understanding of whether, and possibly in what way, chronic Perceived Threat relates to these attributions. We *hypothesized* a replication of the Outgroup Derogation effect observed in Study 1, and in Time 1 of Study 2.

### Time 1 Threat x Time 2 Party Focus on Time 2 Relative Motive Attributions

**Fig 7:**
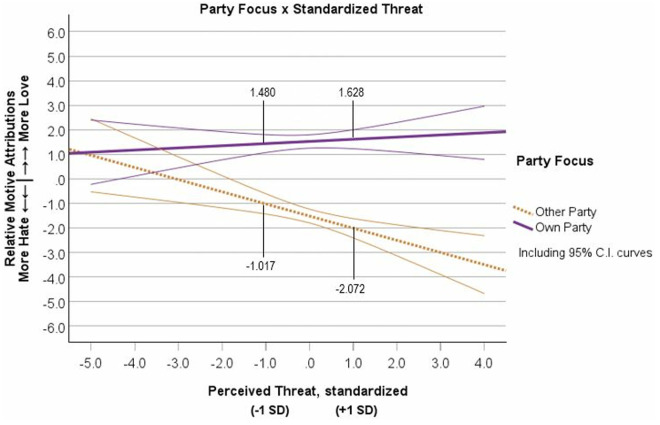
Interaction of Party Focus at Time 2 and Standardized Threat at Time 1 on Time 2 Motive Attributions. Note: Motive Attributions measured as Love minus Hate scores. Error curves show 95% confidence intervals.

Prior to the main analyses, we found that the Time 1 Threat to Time 2 Threat correlation had a strong correlation (*r *= .692, *p* < .001) as expected. Next, we examined Perceived Threat at Time 1 and its relationship to Relative Motive Attributions at Time 2 and tested whether Time 1 Threat interacted with Time 2 Party Focus to predict Time 2 Motive Attributions. We entered effects-coded Party Focus from Time 2, standardized Perceived Threat from Time 1, and its interaction into a regression on Relative Motive Attributions from Time 2. We found a significant effect of Time 1 Threat, *semipartial R*^2^ = .010, *β* = −.082, *t* = −2.522, *p* = .025, *b* = −.227, 95% CI [−.425, −.029] and a significant Time 2 Party Focus x Time 1 Threat interaction, *semipartial R*^2^ = .02, *β* = .109, *t* = 2.985, *p* = .003, *b* = .301, 95% CI [.103,.499]. The Threat simple slope for the Own Party condition was not significant (*R*^2^ = .001, *β* = .027, *p* = .593, *b* = .074, 95% CI [−.174,.353]), but the Threat slope was significant for the Other Party condition (*R*^2^ = .035, *β* = −.191, *p* < .001, *b* = −.527, 95% CI [−.816, −.239]). Again, the interaction pattern supported the Outgroup Derogation effect (see Fig 7). See S9 Appendix I for analyses showing a significant Party x Threat effect when both identification and political orientation are included as covariates in the longitudinal model.

### Discussion

Study 2 examined the relationship between Perceived Threat and Motive Attributions in a longitudinal study. In accordance with Study 1, the Study 2 regression results from Time 1 and Time 2 supported the Outgroup Derogation effect (Hypothesis 2) and failed to support the Ingroup Favoritism effect or the Combined Favoritism-Derogation effect. This Outgroup Derogation pattern was observed consistently in the regression analyses in Study 1 and at both Time 1 and Time 2 of Study 2.

We were also interested in examining this relationship between Perceived Threat and the Motive Attributions over time. Due to the relationship between threat and intergroup bias, we considered only Threat at Time 1 to avoid any possible influence of bias on the threat measure. If the Time 1 Threat x Time 2 Party Focus interaction is like the Time 2 Threat x Time 2 Party Focus interaction, then we have a good idea of the stability of this effect over time. We observed a significant interaction of Time 1 Threat and Time 2 Party Focus (*R*^2^ = .02, *p* = .003) and the pattern replicated both Study 1, and Time 1 Study 2 results in which only the Threat slope in the Other Party condition was significant (*R*^2^ = .03, *p* < .001). Moreover, this Time 1 Threat x Time 2 Party interaction remained significant when including level of social identification and political orientation in the model.

## General discussion

We examined whether Perceived Threat was related to the Motive Attribution Asymmetry Bias (i.e., the Motive Asymmetry Bias) between groups of American Republicans and Democrats. In their original research, Waytz et al. [11] found that groups in conflict make similar attributions about their ingroup’s motives for conflict (i.e., their own group causes conflict by being positive toward their group more than hating the outgroup, but rate the outgroup’s motives as being more about hating their group (our ingroup) than doing positive things for the outgroup). This phenomenon was named the Motive Attribution Asymmetry Bias, or the Motive Asymmetry Bias for short. Our research was the first to investigate how perceived intergroup threat was related to this bias, and we were also able to investigate the separate ingroup favoritism and outgroup derogation components because of the nature of the measurement used. Overall, little research has been done to examine how perceived threat relates to ingroup favoritism as opposed to outgroup derogation for groups in conflict and none when investigating the Motive Asymmetry Bias [26,61,77,78]. The current research sought to test the relationship of perceived threat to ingroup favoritism and outgroup derogation in a Motive Asymmetry Bias paradigm.

Our research focused on American Republicans and Democrats and their level of perceived threat (high or low) from the other political outgroup. We measured both ingroup favoritism and outgroup derogation by having participants rate their ingroup’s motives for conflict with the other political group, or by having them rate the outgroup’s motives for conflict; these ratings were combined into one measure of the Motive Asymmetry Bias. We were the first to replicate the Motive Attribution Asymmetry Bias observed by Waytz and colleagues [11] and we observed similarly large effect sizes (η²_p_ = .258 and η²_p_ = .261). We observed that participants rated their own group’s reasons for causing conflict as being due to positive actions toward their own political group, but not due to negative actions against the outgroup. Participants, however, rated the outgroup’s motives for causing conflict as being due to negative actions against the participant’s ingroup.

When investigating perceived threat, there was a difference for participants who perceived high versus low threat from the political outgroup and whether they had rated the outgroup political party or had rated their own political party. Within our two studies, we observed a Party Focus (Own vs Other group) x Perceived Threat (High vs Low) interaction on Motive Attributions (Love ratings minus Hate ratings). For the Own group rating condition, the Threat simple slope was not significant; thus, perceived threat was not related to how one rated one’s own group on motivations for causing conflict. For the Other group rating condition, however, the Threat simple slope was significant and showed more negative ratings of the Other group for those perceiving higher threat compared to those who perceived lower threat. This pattern of results did not support the Ingroup Favoritism hypothesis or the Combined Favoritism-Derogation hypothesis. Instead, the data indicated that the ratings of group motives were related to only ratings of the other group in that the other group is rated more negatively by those who perceive high threat compared to those who perceive low threat (i.e., an Outgroup Derogation effect). Additionally, political ideology did not moderate this effect; both republicans and democrats showed this outgroup derogation effect (see S1 Table H1 for an overview of the means and standard deviations of variables).

The current research also investigated the relationship of Threat and Motive Asymmetry Bias over time. Within Study 2, we investigated the effect of measured threat over a three-month period and replicated the Party Focus x Threat interaction separately at Time 1 and Time 2. Furthermore, Threat measured at Time 1 interacted with Party Focus at Time 2 to predict Motive Attributions at Time 2 three months later. Again, the same Outgroup Derogation effect was revealed. This longitudinal finding increases our confidence in the cross-sectional results from Study 1 and from Time 1 of Study 2. Given that it was an initial test of these relationships, we used a well-established measure of Perceived Threat, which limited Study 1 to observing correlational relationships for Threat and Motive Attributions. Importantly, we observed a strong correlation between Time 1 Threat and Time 2 Threat (r = .692) and observed that the Time 1 Threat x Time 2 Party Focus interaction remained significant even after accounting for level of social identification and political orientation, both of which could have altered the results; we return to this discussion within the limitations section. Importantly, the longitudinal evidence also suggests that perceived threat could be a chronic factor [58] that could increase the possibility of perceived bias in others in the future and could possibly increase intergroup bias itself. Such a pervasiveness of perceiving threats would be detrimental and is an avenue of research that is worthwhile to pursue.

In both of our studies, we failed to observe a difference for ratings of the ingroup’s motives for conflict between groups in relationship to the perception of high or low threat from the outgroup. We observed only a difference on ratings of the outgroup motivations in which participants who perceived more threat also rated the outgroup’s motives as more negative. In our studies, the idea of threat priming a focus on the outgroup could not be an explanation because we had counterbalanced the measurement of threat and participants had rated both their ingroup and outgroup. Overall, we observed no difference in the pattern when threat was measured before or after the Motive Asymmetry Bias, and we observed Outgroup Derogation in which threat was related to only ratings of the Other group.

### Potential social cognition mechanisms

In their paper in which they provide evidence for the existence of a Motive Attribution Asymmetry Bias, Waytz and colleagues [11] also discuss two potential mechanisms for the bias. While we did not directly test the mechanisms of this bias, we do agree that the two proposed mechanisms are plausible ones for producing the bias. In one mechanism, they propose that favoritism and derogation motives have different cognitive availability for ingroup and outgroup members. Ingroup favoritism may occur more often when people are attempting to affiliate with others, while outgroup derogation may occur more often when people are in competition or expressing anger [79,80]. According to this logic, seeing hate or dislike may be more likely for the outgroup compared to the ingroup. Other research supports a disconnect such as this in the evaluation of groups. When evaluating adversary actions, people may attribute those actions to approach motives (toward the ingroup) more so than to avoidance motives of the outgroup protecting their own group [81]. The second mechanism proposed was one in which people use motivated reasoning to see their ingroup positively and the outgroups negatively. Research had demonstrated an attribution error in which people attribute positive ingroup behaviors to internal group factors and negative ingroup behaviors to external causes. Positive outgroup behaviors, however, are attributed to external causes, while negative outgroup behaviors are attributed to internal factors of the outgroup [82,83]. For the Motive Attribution Asymmetry Bias, only internal motivations (favoritism versus derogation) were being evaluated for the same behavior (of causing conflict), yet we observe a similar pattern of ingroup motives being rated as due to favoritism while outgroup motives are rated as being due to derogation or negativity. Again, we see this mechanism as a plausible one for this bias, and we note that monetary incentives did reduce this bias in the Waytz et al., studies. Research on monetary incentives has shown that this incentive increases people’s willingness to take the perspective of the other party and to be more accurate [84].

We now to turn to other relevant research that sheds some light on the potential mechanism for producing the Threat and Outgroup Derogation effect within our studies. Weisel and Böhm [30] found that outgroup derogation was the “predominant behavioral motivation” (p. 116) for ingroup members’ behaviors when the cost of these behaviors was not blatant harm to the outgroup and the groups in conflict had high animosity toward one another (e.g., supporters of sports teams or supporters of political parties). This may provide some insight into our findings. If ratings of motives in our studies are considered subtle instead of blatant, then it might help to explain why outgroup derogation appears to be a primary mechanism observed in ratings of group motives. In our studies, perceived threat was related to only ratings of the outgroup motives and we showed that higher perceived threat was related to significantly more outgroup derogation in comparison the lower threat. In Weisel and Böhm’s [30] study 2, they used political groups with strong animosity and that were considered morality-based because they differed on principles of right and wrong instead of just attitudes. In the study, these morality-based political groups induced even more outgroup derogation than the groups in their study 1 who experienced only high animosity (supporters of sports teams). Within Study 2, this effect, however, was more reliable and robust when groups had high animosity and moral differences (political groups instead of sports teams); high animosity sports teams and subtle bias was not enough to produce more outgroup derogation than ingroup favoritism for groups without moral differences. Some of these findings match up with findings reviewed by Abramowitz and Webster [31] who showed that the animosity between political parties in the U.S. has been high since the 1980s, but has continued to increase throughout the 2010s. This increasing animosity has occurred in a time of increasing negative partisanship, in which supporters align against one party instead of affiliating with the other [31] and a time of increasing affective polarization in which partisans view each other as disliked outgroups [32]. This negative partisanship and the increasing animosity may drive our outgroup derogation effect even higher for those who perceive more threat from the political outgroup and are able to show subtle bias as opposed to blatant bias. Future research would need to be done to tease apart whether the view of the outgroup drives this effect and whether rating group’s motives for being in conflict are considered subtle versus blatant evaluations; these evaluations may need to interact with animosity and moral difference between groups to produce derogation.

### Limitations

While our study offers promising implications for bias mitigation by highlighting the potential effectiveness of altering perceptions of outgroup motives, it is crucial to acknowledge the limitations of the research. First, the narrow focus of our study on Perceived Threat and Motive Attributions within a specific context (Republicans and Democrats in the United States) may limit the generalizability of our findings to broader populations and to alternative settings. Given that both high animosity and morality-based right versus wrong positions often accompany thinking about these political groups, it will be worthwhile for future research to test these threat effects on other groups with high animosity, but without morality-based positions (e.g., conflict between sports fans such as the Yankees and Red Sox). Second, using a measured predictor brings in its own weaknesses. For the cross-sectional portions of the studies, we were able to only demonstrate correlations between these variables. As a result, we are not able to make causal conclusions and it also allows for the potential for third variables to explain some of the relationships. One such variable could be level of social identification with one’s group creating stronger effects, and which may influence the extent to which individuals are inclined to change their views of the outgroup. A second variable is that of political orientation, which has been shown to be associated with perceptions of symbolic and realistic threat. While these variables may not be the only ones and future research should continue to identify other important ones, we did take steps to test for their influence. To deal with some of these issues, we have attempted to establish temporal precedence with the longitudinal component of Study 2. The significant interaction of Time 1 Threat and Time 2 Party Focus on Motive Attributions helps provide some evidence of threat predicting motives over time. Importantly, we conducted an additional analysis where we included social identification and political orientation in the model, and we still observed a significant Time 1 Threat x Time 2 Party Focus interaction. While these analyses do not alleviate all concerns, they do help bolster the findings. Future research can take the next step in this line by manipulating threat to demonstrate causal relationships. Despite these limitations, our findings underscore the potential of interventions targeting perceptions of outgroup motives as a viable strategy for mitigating bias, suggesting avenues for further research to explore and refine such interventions in diverse contexts.

### Implications

These studies show promising implications for bias mitigation strategies, particularly in the realm of intergroup relations with groups in conflict. Perceived Threat explained substantial variance across our studies when examining motives of the other party. These findings suggest that a focus on outgroup motives and perceptions could be one way to reduce bias and highlight a practical avenue for promoting positive intergroup dynamics. Given that favoritism and derogation motives may have different cognitive availability for ingroups and outgroups, changing these availabilities may be an avenue to changing perceptions of the outgroup’s motives for engaging in conflict [79–81]. In previous research, providing a financial incentive helped participants see the other group’s motives more accurately [11]. While this may not be a viable large-scale strategy due to cost, there may be other strategies that can encourage people to think about alternative causes for conflict that do not come to mind easily.

Such insights may be drawn from literature around peace psychology, and conflict resolution and negotiation research. Praszkier et al. [85] drew on literature around peace psychology to investigate measures of a peace-oriented mindset, which is a mindset that may encourage change in perceptions about others. One of their findings was that involvement in social activities is related to this peace-oriented mindset. It is well-established in the literature that intergroup contact serves as a facilitator for improving intergroup relations [86–90]. So, providing group social activities may be another way to shift perceptions of outgroup motives by fostering this peace-oriented mindset, through intergroup contact. Within negotiation research, those in a value driven conflict are engaged in one where the fundamental values of either party may not necessarily be incompatible, but within the conflict, these values are perceived to be incompatible by one or both parties [91]. This is analogous to the literature cited earlier around asymmetry in perception of motives, the motives themselves are not necessarily incompatible, but they are seen to be different by one or both parties. Interventions provided to negotiating parties which promoted an integrated mindset – an inclination to collaborate, be curious, be creative – in their approach to negotiation were shown to respect their counterpart more than in the control condition [91]. Such interventions promoting collaboration, curiosity, and creativity may be altered to other intergroup scenarios related more specifically to conflict to alter perception and improve intergroup relations. The current research also suggests that interventions aimed at addressing perceptions of threat from the outgroup could also yield enduring effects. Ratings of the causes of conflict for one’s own group were unchanged in relation to perceived threat and showed that ingroup favoritism persisted. This persistence of ingroup favoritism would still cause problems for intergroup dialogs and negotiations. Thus, while these intervention ideas with focus on the outgroup are a good place to begin, further research is still needed to understand any interplay between the unchanging nature of a positive ingroup view and altering perceptions of the outgroup. Solutions to changing perceptions of motives would need to be multifaceted in which interventions would both lessen threat to reduce negative perceptions of the outgroup and focus on perceptions of the outgroup relating to perceptions of the ingroup.

## Conclusion

Previous research indicates that there is a relationship between threat and intergroup bias, with higher threat relating to more intergroup bias. Perceived threat from an outgroup does increase intergroup bias, which provides a point from which to investigate and to further help improve relations between and among groups. The current research extended this research into threat and intergroup bias by examining how Perceived Threats are related to the Motive Attribution Asymmetry Bias and the motives people use to explain why their ingroups and outgroups are causing conflict with one another. We consistently found support for the existence of a large Motive Attribution Asymmetry Bias effect, and we found that perceived threat was related to a larger bias. When examining this effect, we discovered that perceived threat was related to only outgroup ratings and not to ingroup ratings. Overall, higher perceived threat was related to more negative motive ratings of the Other group (Outgroup Derogation Effect), but it was not related to ratings of the Own group (Ingroup Favoritism). This is an unusual finding given that much research finds that intergroup bias occurs because groups are favoring their ingroups instead of derogating or showing hostility toward outgroups, even when in conflict. There appear to be a few key factors that may produce this difference. More outgroup derogation is often, though not always, observed with groups with high animosity toward one another, and it may require animosity being paired with moral differences of right and wrong between groups. Second, outgroup derogation may be more likely when the derogation is subtle instead of blatant. It may be that rating motives for conflict is a subtle form of bias, which allows people to make more negative ratings of an outgroup; our finding matches with results observed for groups in conflict experiencing subtle versus blatant harm to the outgroup where more derogation of the outgroup occurred when harm was subtle. Future research could build on these findings to examine this possibility and to help lessen bias and improve intergroup relations.

## Supporting information

S1 Table Overview of Means and SDs for all Studies. (DOCX)

S1 Appendix Perceived Threat from the Other Party Scale. (DOCX)

S2 Appendix Motive Attributions Scale. (DOCX)

S3 Appendix Short Form of the Need for Cognition Scale. (DOCX)

S4 Appendix Measures of Political Affiliation. (DOCX)

S5 Appendix Demographics and Filler Questions. (DOCX)

S6 Appendix Social Identification Measure. (DOCX)

S7 Appendix Correlations Among Variables of Interest. (DOCX)

S8 Appendix Analyses testing for Order of variable effects and for testing interaction with Political Orientation. (DOCX)

S9 Appendix Analyses with Social Identification. (DOCX)

S10 Appendix Separate Analyses for Symbolic Threat and for Realistic Threat. (DOCX)
