# Supplementary material for: The influence of perceived threat on the motive attribution asymmetry bias for groups in conflict
Source: PLoS One. 2025 Sep 4;20(9):e0330927. doi: 10.1371/journal.pone.0330927 (PMC12410775; doi:10.1371/journal.pone.0330927)
Supplement: S1 Table — (DOCX) [file pone.0330927.s001.docx]

Table H1 - Overview of Means and SDs for all Studies

|  |  | Motive Attributions | | Perceived Threat | | Motive Attributions by Threat | |
| --- | --- | --- | --- | --- | --- | --- | --- |
|  |  | Mean | SD | Mean | SD | Low Threat (-1 SD) | High Threat (+1 SD) |
| Study 1 | Own Party Focus | 1.107 | 2.146 | 5.060 | 0.806 | 1.144 | 1.071 |
|  | Other Party Focus | -1.658 | 2.551 | 5.036 | 0.822 | -0.502 | -2.849 |
| Study 2, T1 | Own Party Focus | 1.222 | 0.912 | 4.987 | 1.977 | 1.153 | 1.243 |
|  | Other Party Focus | -1.463 | 0.843 | 5.069 | 2.342 | -0.339 | -2.215 |
| Study 2, T2 | Own Party Focus | 1.553 | 0.870 | 5.032 | 2.185 | 1.509 | 1.597 |
|  | Other Party Focus | -1.584 | 0.793 | 5.049 | 2.345 | -0.753 | -2.391 |
| Study 2: Threat T1, Motive Attributions T2 | Own Party Focus | 1.553 | 0.870 | 4.987 | 1.977 | 1.480 | 1.628 |
|  | Other Party Focus | -1.584 | 0.793 | 5.069 | 2.342 | -1.018 | -2.072 |
