# Supplementary material for: The influence of perceived threat on the motive attribution asymmetry bias for groups in conflict
Source: PLoS One. 2025 Sep 4;20(9):e0330927. doi: 10.1371/journal.pone.0330927 (PMC12410775; doi:10.1371/journal.pone.0330927)
Supplement: S1 Appendix — (DOCX) [file pone.0330927.s002.docx]

**Appendix A**

**Perceived Threat from the Other Party Scale**

1. The other party holds too many positions of power and responsibility in this country.

- Disagree Strongly
- Disagree Moderately
- Disagree Somewhat
- Neither Agree nor Disagree
- Agree Somewhat
- Agree Moderately
- Agree Strongly

1. The other party dominate American politics more than they should.
2. When the other party is in positions of authority, they discriminate against my party when making hiring decisions.
3. Too much money is spent on educational programs that benefit the other party.
4. The other party has more economic power than they deserve in this country.
5. The other party receives too much of the money spent on healthcare and childcare.
6. Too little money per student is spent on education for the other party.
7. The tax system favors the other party.
8. Many companies hire less qualified members of the other party over more qualified members of my party.
9. The other party has more political power than they deserve in this country.
10. Public service agencies favor the other party over my party.
11. The legal system is more strict on the other party than on my party.
12. My party has very different values than the other party.
13. The other party has no right to think they have better values than my party.
14. The other party wants their rights to be put ahead of the rights of my party.
15. The other party doesn’t understand the way my party views the world.
16. The other party does not value the rights granted by the Constitution (life, liberty, and the pursuit of happiness) as much as my party does.
17. The other party and my party have different family values.
18. The other party doesn’t value the traditions of their party as much as my party does.
19. The other party regard themselves as morally superior to my party.
20. The values of the other party regarding work are different from those of my party.
21. Most members of the other party will never understand what members of my party are like.
22. The other party should not try to impose their values on my party.
23. My party does not get as much respect from the other party as they deserve.
