# Supplementary material for: The influence of perceived threat on the motive attribution asymmetry bias for groups in conflict
Source: PLoS One. 2025 Sep 4;20(9):e0330927. doi: 10.1371/journal.pone.0330927 (PMC12410775; doi:10.1371/journal.pone.0330927)
Supplement: S2 Appendix — (DOCX) [file pone.0330927.s003.docx]

**Appendix B**

**Motive Attributions Scale**

Rate Your Own Party – Positive Motives

- When your party engages in conflict, how much is your party motivated by empathy towards your political party?
- 1 – Not at all
- 2
- 3
- 4 – Moderately
- 5
- 6
- 7 – Very Much
- When your party engages in conflict, how much is your party motivated by compassion towards your political party?
- When your party engages in conflict, how much is your party motivated by kindness towards your political party?

Rate Your Own Party – Negative Motives

- When your party engages in conflict, how much is your party motivated by hatred towards the other party?
- When your party engages in conflict, how much is your party motivated by dislike towards the other party?
- When your party engages in conflict, how much is your party motivated by disdain towards the other party?

Rate the Other Party – Positive Motives

- When the other party engages in conflict, how much is their party motivated by empathy towards their political party?
- When the other party engages in conflict, how much is their party motivated by compassion towards their political party?
- When the other party engages in conflict, how much is their party motivated by kindness towards their political party?

Rate the Other Party – Negative Motives

- When the other party engages in conflict, how much is their party motivated by hatred towards your party?
- When the other party engages in conflict, how much is their party motivated by dislike towards your party?
- When the other party engages in conflict, how much is their party motivated by disdain towards your party?
