# Supplementary material for: The influence of perceived threat on the motive attribution asymmetry bias for groups in conflict
Source: PLoS One. 2025 Sep 4;20(9):e0330927. doi: 10.1371/journal.pone.0330927 (PMC12410775; doi:10.1371/journal.pone.0330927)
Supplement: S3 Appendix — (DOCX) [file pone.0330927.s004.docx]

**Appendix C**

**Short Form of the Need for Cognition Scale**

Instructions: For each of the statements below, please indicate to what extent the statement is characteristic of you. If the statement is extremely uncharacteristic of you (not at all like you) please write a "1" to the left of the question; if the statement is extremely characteristic of you (very much like you) please write a "5" next to the question. Of course, a statement may be neither extremely uncharacteristic nor extremely characteristic of you; if so, please use the number in the middle of the scale that describes the best fit. Please keep the following scale in mind as you rate each of the statements below: 1 = extremely uncharacteristic; 2 = somewhat uncharacteristic; 3 = uncertain; 4 = somewhat characteristic; 5 = extremely characteristic.

1. I would prefer complex to simple problems.

2. I like to have the responsibility of handling a situation that requires a lot of thinking.

3. Thinking is not my idea of fun. a

4. I would rather do something that requires little thought than something that is sure to challenge my thinking abilities?

5. I try to anticipate and avoid situations where there is a likely chance I will have to think in depth about something."

6. I find satisfaction in deliberating hard and for long hours.

7. I only think as hard as 1 have to. a

8. I prefer to think about small, daily projects to long-term ones?

9. I like tasks that require little thought once I've learned them?

10. The idea of relying on thought to make my way to the top appeals to me.

1 I. I really enjoy a task that involves coming up with new solutions to problems.

12. Learning new ways to think doesn't excite me very much?

13. I prefer my life to be filled with puzzles that I must solve.

14. The notion of thinking abstractly is appealing to me.

15. I would prefer a task that is intellectual, difficult, and important to one that is somewhat important but does not require much thought.

16. 1 feel relief rather than satisfaction after completing a task that required a lot of mental effort?

17. It's enough for me that something gets the job done; I don't care how or why it works?

18. I usually end up deliberating about issues even when they do not affect me personally.

*Note.* From "'The Efficient Assessment of Need for Cognition," by J. T. Cacioppo, R. E. Petty, and C. F. Kao, 1984, *Journal of Personality Assessment, 48,* pp. 306-307. Copyright 1984 by Lawrence Erlbaum. The number of response options on the scales used across studies has typically ranged from five to nine, and the labels for these response options have varied from agreement—disagreement to extremely uncharacteristic-extremely characteristic. Although these variations across studies may influence the total scores obtained, they have not had dramatic effects on the relationships between interindividual variations in need for cognition and other variables in a given study.

a Reverse scored.
