# Supplementary material for: The influence of perceived threat on the motive attribution asymmetry bias for groups in conflict
Source: PLoS One. 2025 Sep 4;20(9):e0330927. doi: 10.1371/journal.pone.0330927 (PMC12410775; doi:10.1371/journal.pone.0330927)
Supplement: S4 Appendix — (DOCX) [file pone.0330927.s005.docx]

**Appendix D**

**Measures of Political Affiliation**

Political Alignment

What political party do you feel more closely aligned with?

- Republican
- Democrat

Political Orientation

Please rate your, personal political orientation

- Extremely Liberal
- Moderately Liberal
- Somewhat Liberal
- Moderate
- Somewhat Conservative
- Moderately Conservative
- Extremely Conservative
