# Supplementary material for: The influence of perceived threat on the motive attribution asymmetry bias for groups in conflict
Source: PLoS One. 2025 Sep 4;20(9):e0330927. doi: 10.1371/journal.pone.0330927 (PMC12410775; doi:10.1371/journal.pone.0330927)
Supplement: S5 Appendix — (DOCX) [file pone.0330927.s006.docx]

**Appendix E**

**Demographics and Filler Questions**

Demographics

Please indicate your gender by using the mouse to click on the appropriate box.

- Female
- Male

Please indicate your race or ethnicity.

- Southeast Asian
- White
- African American/Afro-Caribbean
- Middle Eastern or North African
- Pakistani
- Indian or Indian Sub-continent
- Native American
- Pacific Islander
- Latino or Hispanic
- Other

Please type in your age

In what country do you live?

- USA
- UK
- Other

Filler Questions

Mathematics Questions: 50 x 7 = ____; 13 + 24 = ___
