# Supplementary material for: The influence of perceived threat on the motive attribution asymmetry bias for groups in conflict
Source: PLoS One. 2025 Sep 4;20(9):e0330927. doi: 10.1371/journal.pone.0330927 (PMC12410775; doi:10.1371/journal.pone.0330927)
Supplement: S6 Appendix — (DOCX) [file pone.0330927.s007.docx]

**Appendix F**

**Social Identification Measure**

I identify strongly with other people in my political party.

Being a member of my political party is an important part of who I am.

I feel strong ties with other people in my political party.

I feel a sense of solidarity with other people in my political party.

Disagree Strongly

Disagree Moderately

Disagree Somewhat

Neither disagree or agree

Agree Somewhat

Agree Moderately

Agree Strongly
