# Supplementary material for: The influence of perceived threat on the motive attribution asymmetry bias for groups in conflict
Source: PLoS One. 2025 Sep 4;20(9):e0330927. doi: 10.1371/journal.pone.0330927 (PMC12410775; doi:10.1371/journal.pone.0330927)
Supplement: S7 Appendix — (DOCX) [file pone.0330927.s008.docx]

Appendix G

Correlations Among Variables of Interest

Table G1 - Study 1

|  | Love | Hate | Symbolic Threat | Realistic Threat | Perceived Threat |
| --- | --- | --- | --- | --- | --- |
| Hate | -.321** |  |  |  |  |
| Symbolic Threat | -.026 | .328** |  |  |  |
| Realistic Threat | -.015 | .233** | .683** |  |  |
| Perceived Threat | -.022 | .303** | .908** | .927** |  |
| Motive Attributions | .812** | -.814** | -.218** | -.153** | -.200** |

*Note: ** Correlation significant at the 0.01 level*

*Perceived Threat calculated as mean of Symbolic and Realistic Threat scores*

*Motive Attributions calculated as Love Score – Hate Score*

Table G2 - Study 2, Time 1

|  | Love | Hate | Symbolic Threat | Realistic Threat | Perceived Threat |
| --- | --- | --- | --- | --- | --- |
| Hate | -.182** |  |  |  |  |
| Symbolic Threat | .068 | .336** |  |  |  |
| Realistic Threat | .034 | .268** | .706** |  |  |
| Perceived Threat | .054 | .325** | .915** | .931** |  |
| Motive Attributions | .754** | -.783** | -.181** | -.158** | -.183** |

*Note: ** Correlation significant at the 0.01 level*

*Perceived Threat calculated as mean of Symbolic and Realistic Threat scores*

*Motive Attributions calculated as Love Score – Hate Score*

Table G3 - Study 2, Time 2

|  | Love | Hate | Symbolic Threat | Realistic Threat | Perceived Threat |
| --- | --- | --- | --- | --- | --- |
| Hate | -.277** |  |  |  |  |
| Symbolic Threat | .103* | .288** |  |  |  |
| Realistic Threat | .054 | .247** | .678** |  |  |
| Perceived Threat | .084 | .291** | .910** | .921** |  |
| Motive Attributions | .789** | -.809** | -.121** | -.125** | -.134** |

*Note: ** Correlation significant at the 0.01 level*

** Correlation significant at the 0.05 level*

*Perceived Threat calculated as mean of Symbolic and Realistic Threat scores*

*Motive Attributions calculated as Love Score – Hate Score*
