# Supplementary material for: The influence of perceived threat on the motive attribution asymmetry bias for groups in conflict
Source: PLoS One. 2025 Sep 4;20(9):e0330927. doi: 10.1371/journal.pone.0330927 (PMC12410775; doi:10.1371/journal.pone.0330927)
Supplement: S8 Appendix — (DOCX) [file pone.0330927.s009.docx]

Appendix H

**Analyses testing for Order of variable effects and for testing interaction with Political Orientation in the Time 1 and Time 2 data of Study 2.**

**Party Focus x Threat x Order of Variables analyses:**

*Party Focus x Threat x Order of Variables analyses at Time 1 of Study 2.*

As in Study 1, we tested for order effects in Study 2 at Time 1 by conducting a regression with effects-coded Party Focus, effects-coded Order (i.e., Order of Threat and Party Focus variables), standardized Perceived Threat, and all interaction terms. Again, we found a non-significant Party Focus x Threat x Order interaction, *R*^2^ < .001, *β* = -.01, *t =* -.297, *p* = .766, *b* = -.024, 95% CI [-.185, .136], a non-significant Party Focus x Order interaction, *R*^2^ < .001, *β* = .022, *t* = .667, *p* = .505, *b* = .054, 95% CI [-.105, .212], and a non-significant Threat x Order interaction, *R*^2^ = .001, *β* = .024, *t* = .715, *p* = .475, *b* = .058, 95% CI [-.102, .219]. These analyses indicated that order of presentation did not significantly change participant’s responses.

*Party Focus x Threat x Order of Variables analyses at Time 2 of Study 2.*

We conducted a regression with effects-coded Party Focus, effects-coded Order (i.e., Order of Threat and Party Focus), standardized Perceived Threat, and all interaction terms on Time 2 data. Again, we found a non-significant Party Focus x Threat x Order interaction, *R*^2^ = .001, *β* = -.018, *t* = -.503, *p* =.615, *b* = -.050, 95% CI [-.246, .146], and non-significant Party Focus x Order, *R*^2^ < .001, *β* = .005, *t* = .147, *p* = .883, *b* = .014, 95% CI [-.180, .208] and Threat x Order interactions, *R*^2^ = .002, *β* = -.038, *t* = -1.062, *p* = .289, *b* = -.106, 95% CI [-.301, .090]. These analyses indicated that order of presentation did not significantly influence responses.

**Party Focus x Threat x Political Orientation analyses:**

*Party Focus x Threat x Political Orientation analyses at Time 1 of Study 2.*

To test whether Political Orientation interacted with the main Study 2 variables at Time 1, we entered effects-coded Party Focus, standardized Political Orientation, standardized Perceived Threat, and their interactions into a regression on Motive Attributions. We observed a non-significant Party Focus x Threat x Political Orientation interaction, *R*^2^ = .002, *β* = -.037, *t* = -1.082, *p* = .280, *b* = -.085, 95% CI [-.238, .069]. Thus, political orientation did not qualify the main results. The Party Focus x Threat interaction remained significant, *R*^2^ = .054, *β* = .200, *t* = 5.996, *p* < .001, *b* = .495, 95% CI [.333, .657], as did the Threat main effect, *R*^2^ = .045. *β* = -.182, *t* = -5.463, *p* < .001, *b* = -.451, 95% CI [-.613, -.289], and the Party Focus main effect *R*^2^ = .282, *β* = .508, *t* = 15.763, *p* < .001, *b* = 1.256, 95% CI [1.099, 1.412]. Thus, Political Orientation did not moderate the effects.

*Party Focus x Threat x Political Orientation analyses at Time 2 of Study 2.*

We entered effects-coded Party Focus, standardized Political Orientation, standardized Perceived Threat, and their interactions into a regression on Motive Attributions to test whether Political Orientation interacted with the main variables. We observed a non-significant Party Focus x Threat x Political Orientation interaction, *R*^2^ < .001, *β* = -.007, *t* = -.184, *p* = .854, *b* = -.018, 95% CI [-.210, .174]. Thus, Political Orientation did not qualify the main results of the Motive Attributions. The Party Focus x Threat interaction remained significant, *R*^2^ = .036, *β* = .161, *t* = 4.299, *p* < .001, *b* = .444, 95% CI [.241, .647], as did the main effect of Threat, *R*^2^ = .022, *β* = -0.125, *t* = -3.327, *p* < .001, *b* = -.344, 95% CI [-.547, -.141], and main effect of Party Focus, *R*^2^ = .344, *β* = 0.571, *t* = 16.062, *p* < .001, *b* = 1.573, 95% CI [1.381, 1.766]. Participants’ Political Orientation did not qualify any of the results we found on the relationship between Threat, Party Focus, and Motive Attributions.
