# Supplementary material for: The influence of perceived threat on the motive attribution asymmetry bias for groups in conflict
Source: PLoS One. 2025 Sep 4;20(9):e0330927. doi: 10.1371/journal.pone.0330927 (PMC12410775; doi:10.1371/journal.pone.0330927)
Supplement: S9 Appendix — (DOCX) [file pone.0330927.s010.docx]

Appendix I

Analyses with Social Identification

**Time 2 Party Focus x Time 1 Threat interaction with Time 2 covariates of Social Identification and Political Orientation in the model**.

To test for the effects with covariates in the model, we entered Time 2 Effects-coded Party Focus, Time 1 standardized Threat, Time 2 Social Identification, Time 2 Political orientation, and the Time 2 Party Focus x Time 1 Threat interaction into the model. With the Time 2 covariates of Social Identification and Political Orientation in the model, we once again observed a significant Time 2 Party Focus x Time 1 Threat interaction, *R*^2^ = .02, *β* = .104, *t* = 2.874, *p* = .004, *b* = .287, 95% CI [.091, .483].

**Time 2 Party Focus x Time 1 Threat interaction with Time 2 covariates of Threat, Social Identification, and Political Orientation in the model**.

We entered Time 2 Effects-coded Party Focus, Time 1 standardized Threat, Time 2 Threat, Time 2 Social Identification, Time 2 Political orientation, and the Time 2 Party Focus x Time 1 Threat interaction into the model. With the Time 2 covariates of Threat, Social Identification, and Political Orientation in the model, we still observed a significant Time 2 Party Focus x Time 1 Threat interaction, *R*^2^ = .02, *β* = .103, *t* = 2.880, *p* = .004, *b* = .284, 95% CI [.090, .478]. This analysis shows that the main Party x Threat interaction remained significant even after controlling for the influence of T2 Threat, T2 Social Identification, and T2 Political Orientation.
