# Supplementary material for: The influence of perceived threat on the motive attribution asymmetry bias for groups in conflict
Source: PLoS One. 2025 Sep 4;20(9):e0330927. doi: 10.1371/journal.pone.0330927 (PMC12410775; doi:10.1371/journal.pone.0330927)
Supplement: S10 Appendix — (DOCX) [file pone.0330927.s011.docx]

Appendix J

**Analyses testing Party Focus x Symbolic Threat, and Party Focus x Realistic Threat on Relative Motive Attributions in Study 1 and Study 2.**

**Party Focus x Symbolic Threat analyses:**

*Party Focus x Symbolic Threat analyses in Study 1.*

We conducted a regression with effects-coded Party Focus, standardized Symbolic Perceived Threat, and all interaction terms. The analysis revealed a significant main effect of standardized Symbolic Threat, _s_*r*^2^ = .066, *β* = -.214, *t* = -6.678, *p* < .001, *b* = -.580, 95% CI [--.751, -.410], and a main effect of Party Focus, _s_*r*^2^ = .285, *β* = .508, *t* = 15.856, *p* < .001, *b* = 1.382, 95% CI [1.210, 1.553] on Motive Attributions. These main effects were qualified by a significant Party Focus x Symbolic Threat interaction, _s_*r*^2^ = .069, *β* = .219, *t* = 6.850, *p* < .001, *b* = .595, 95% CI [.425, .766].

*Party Focus x Symbolic Threat analyses in Study 2.*

We conducted a regression with effects-coded Party Focus, standardized Symbolic Perceived Threat, and all interaction terms. The analysis revealed a significant main effect of standardized Symbolic Threat, _s_*r*^2^ = .041, *β* = -.169, *t* = -5.198, *p* < .001, *b* = -.418, 95% CI [-.576, -.260], and a main effect of Party Focus, _s_*r*^2^ = .275, *β* = .504, *t* = 15.528, *p* < .001, *b* = 1.247, 95% CI [1.089, 1.404] on Motive Attributions. These main effects were qualified by a significant Party Focus x Symbolic Threat interaction, _s_*r*^2^ = .058, *β* = .203, *t* = 6.260, *p* < .001, *b* = .503, 95% CI [.346, .661].

**Party Focus x Realistic Threat analyses:**

*Party Focus x Realistic Threat analyses in Study 1.*

We conducted a regression with effects-coded Party Focus, standardized Realistic Perceived Threat, and all interaction terms. The analysis revealed a significant main effect of standardized Realistic Threat, _s_*r*^2^ = .047, *β* = -.193, *t* = -5.759, *p* < .001, *b* = -.522, 95% CI [-.701, -.34], and a main effect of Party Focus, _s_*r*^2^ = .277, *β* = .513, *t* = 15.549, *p* < .001, *b* = 1.397, 95% CI [1.221, 1.574] on Motive Attributions. These main effects were qualified by a significant Party Focus x Realistic Threat interaction, _s_*r*^2^ = .04, *β* = .167, *t* = 4.984, *p* < .001, *b* = .452, 95% CI [.274, .630].

*Party Focus x Realistic Threat analyses in Study 2.*

We conducted a regression with effects-coded Party Focus, standardized Realistic Perceived Threat, and all interaction terms. The analysis revealed a significant main effect of standardized Realistic Threat, _s_*r*^2^ = .037, *β* = -.160, *t* = -4.857, *p* < .001, *b* = -.395, 95% CI [-.554, -.235], and a main effect of Party Focus, _s_*r*^2^ = .275, *β* = .510, *t* = 15.513, *p* < .001, *b* = 1.260, 95% CI [1.100, 1.419] on Motive Attributions. These main effects were qualified by a significant Party Focus x Realistic Threat interaction, _s_*r*^2^ = .036, *β* = .159, *t* = 4.849, *p* < .001, *b* = .394, 95% CI [.235, .554].
